# New structural analogues of curcumin exhibit potent growth suppressive activity in human colorectal carcinoma cells

**DOI:** 10.1186/1471-2407-9-99

**Published:** 2009-03-30

**Authors:** Ling Cen, Brian Hutzen, Sarah Ball, Stephanie DeAngelis, Chun-Liang Chen, James R Fuchs, Chenglong Li, Pui-Kai Li, Jiayuh Lin

**Affiliations:** 1 Department of Pediatrics, The Ohio State University, Columbus, Ohio, 43210, USA; 2 Molecular Cellular and Developmental Biology Program, The Ohio State University, Columbus, Ohio, 43210, USA; 3 Division of Medicinal Chemistry and Pharmacognosy, College of Pharmacy, The Ohio State University, Columbus, Ohio, 43210, USA; 4 Experimental Therapeutics Program, The Ohio State University Comprehensive Cancer Center, College of Medicine, The Ohio State University, Columbus, Ohio, 43210, USA; 5 Center for Childhood Cancer, The Research Institute at Nationwide Children's Hospital, Department of Pediatrics, College of Medicine, The Ohio State University, 700 Children's Drive, Columbus, OH 43205, USA

## Abstract

**Background:**

Colorectal carcinoma is one of the major causes of morbidity and mortality in the Western World. Novel therapeutic approaches are needed for colorectal carcinoma. Curcumin, the active component and yellow pigment of turmeric, has been reported to have several anti-cancer activities including anti-proliferation, anti-invasion, and anti-angiogenesis. Clinical trials have suggested that curcumin may serve as a potential preventive or therapeutic agent for colorectal cancer.

**Methods:**

We compared the inhibitory effects of curcumin and novel structural analogues, GO-Y030, FLLL-11, and FLLL-12, in three independent human colorectal cancer cell lines, SW480, HT-29, and HCT116. MTT cell viability assay was used to examine the cell viability/proliferation and western blots were used to determine the level of PARP cleavages. Half-Maximal inhibitory concentrations (IC_50_) were calculated using Sigma Plot 9.0 software.

**Results:**

Curcumin inhibited cell viability in all three of the human colorectal cancer cell lines studied with IC_50 _values ranging between 10.26 μM and 13.31 μM. GO-Y030, FLLL-11, and FLLL-12 were more potent than curcumin in the inhibition of cell viability in these three human colorectal cancer cell lines with IC_50 _values ranging between 0.51 μM and 4.48 μM. In addition, FLLL-11 and FLLL-12 exhibit low toxicity to WI-38 normal human lung fibroblasts with an IC-50 value greater than 1,000 μM. GO-Y030, FLLL-11, and FLLL-12 are also more potent than curcumin in the induction of apoptosis, as evidenced by cleaved PARP and cleaved caspase-3 in all three human colorectal cancer cell lines studied.

**Conclusion:**

The results indicate that the three curcumin analogues studied exhibit more potent inhibitory activity than curcumin in human colorectal cancer cells. Thus, they may have translational potential as chemopreventive or therapeutic agents for colorectal carcinoma.

## Background

In the United States, colorectal cancer is the third most frequently occurring cancer in both sexes and overall is the second leading cause of cancer deaths. The lifetime probability of developing colorectal cancer is about 5% in the United States. Despite advances in the treatment of colorectal cancer, the five-year survival rate has only increased to 65% [1]. Hence, better approaches for the prevention and treatment of colorectal cancer are needed.

Curcumin has been shown to protect against carcinogenesis and to prevent tumor formation and development in several cancer types. It has also been shown to suppress angiogenesis and metastasis in a variety of animal tumor models [2-6]. Curcumin is a bioactive component found in the rhizome of the perennial herb *Curcuma longa*. A polyphenolic compound with intense yellow coloring, curcumin has been part of therapeutic preparations for centuries due to its wide spectrum of beneficial activities and its safety in relatively large doses [7]. Extensive research has indicated that the complex chemistry of curcumin allows it to influence multiple cell signaling pathways, giving it anti-inflammatory, antioxidant, chemopreventive, and chemotherapeutic properties in addition to many others [8]. The anti-carcinogenic properties of curcumin continue to be a subject of great interest. Evidence that it can inhibit the initiation, progression, and continued survival of cancerous cells, likewise, continues to accumulate [8]. Curcumin inhibits cell proliferation by interfering with the cell cycle and inducing apoptosis in colorectal carcinoma cells [9,10]. Curcumin also has chemopreventive potential for colorectal cancer as seen in a mouse model and in human clinical trials [4,8,11,12].

Despite promising findings, curcumin has yet to be approved as an effective chemotherapeutic agent. Testing in animal models and human clinical trials has revealed that the bioavailability of curcumin is low, owing to its poor absorption across the gut, limited tissue distribution, rapid metabolism, and its subsequent elimination from the body [13]. In light of these findings, numerous strategies have been devised to address the limitations of curcumin, including the design and synthesis of novel structural analogues [14]. One such compound is GO-Y030 [15] and two other compounds, FLLL-11 and FLLL-12, which were synthesized by our laboratories. In our present study, we compared the inhibitory efficacy of GO-Y030, FLLL-11, FLLL-12, and curcumin in human colorectal cancer cell lines. We demonstrated that GO-Y030, FLLL-11, and FLLL-12 are more active than curcumin in the inhibition of cell proliferation and induction of PARP and caspase-3 cleavages in all three colorectal cancer cell lines. GO-Y030 appears to be slightly more potent than FLLL-11 and FLLL-12 in these colorectal cancer cell lines. Therefore, the synthetic derivatives of curcumin studied have potential as new therapeutic agents for colorectal cancer.

## Methods

### Cell Culture

Human colorectal cancer cells, HCT-116, SW480, and HT-29, WI-38 human lung fibroblasts, and MCF-10A immortalized human mammary epithelial cells were acquired from the American Type Culture Collection (ATCC). The cancer cells were maintained in 1× Dulbecco's Modification of Eagle's Medium (DMEM) supplemented with 10% fetal bovine serum (FBS) (Invitrogen), 4.5 g/L L-glutamine and sodium pyruvate (Mediatech), and 1% Penicillin/Streptomycin (P/S). The cells were kept in a humidified 37°C incubator and aired with 5% CO_2_. The MCF-10A cells were maintained in Ham's F12 media (Mediatech) supplemented with 5% FBS, 5 μg/ml insulin, 1 μg/ml hydrocortisone, 10 μg/ml epidermal growth factor (EGF), 100 μg/ml cholera toxin, and 1% P/S. Normal human bladder smooth muscle cells (HdSMC) were purchased from Lonza (Walkersville, MD) and Normal human colonic smooth muscle cells (HCSMC) were purchased from ScienCell (Carlsbad, CA).

### Western blot analysis

HCT-116, SW480, and HT-29 colorectal cancer cells, WI-38, HdSMC, HCSMC, and MCF-10A cells were treated with the listed concentrations of GO-Y030, FLLL-11, FLLL-12, and Curcumin (Sigma-Aldriich, St. Louis, MO) for 24 hours. Total protein lysates (50 μg/lane), as determined by BCA Protein Assay Kit (Thermo Fisher Scientific, Rockford, IL), were separated by 10% SDS-polyacrylamide gels and transferred to membranes. Membranes were blotted with a 1:1000 dilution of antibodies against cleaved Poly (ADP-ribose) polymerase (PARP) (Asp214) and cleaved caspase-3 (Asp175) (Cell Signaling Technology, Beverly, MA). A 1:1000 dilution of anti-GAPDH (glyceraldehydes-3-phosphoate dehydrogenase) monoclonal antibody (MAB374, Chemicon International, Inc., Temecula, CA) was used as a protein loading control. The blots were incubated with a 1:600 dilution of secondary fluorescein-linked anti-mouse or anti-rabbit antibody followed by incubation with a 1:2500 dilution of alkaline phosphatase conjugated anti-fluorescein antibody (Amersham Biosciences, Piscataway, NJ). Blots were scanned with ImageQuant software using an ECF Western blotting detection system (Amersham Biosciences, Piscataway, NJ) on a Molecular Dynamics Storm PhosphorImager (Sunnyvale, CA). The fluorescent signals were scanned and documented using a Storm 860 scanner (Molecular Dynamics, Sunnyvale, CA, USA).

### MTT Cell Viability Assay

HCT-116, SW480, and HT-29 human colorectal cancer cells and WI-38 lung fibroblasts were seeded in 96-well plates (4000 cells/well) in DMEM with 10% FBS. The following day the cells were treated with GO-Y030, FLLL-11, FLLL-12, and Curcumin (Sigma-Aldriich, St. Louis, MO) as indicated and incubated for 72 hours. 25 μl of MTT (Thiazolyl Blue Tetrazolium Bromide: M5655, SIGMA) was added to each well and incubated for 3.5 hours followed by the addition of 100 μl of N,N-dimethylformamide (D4551, SIGMA) solubilization solution. The plates were left at room temperature overnight to allow complete lysis of the cells and read at 450 nm the following day. Half-Maximal inhibitory concentrations (IC_50_), the drug concentration at which 50% growth inhibition is achieved, was calculated using Sigma Plot 9.0 software (Systat Software Inc., San Jose, CA) with the 4 parameter logistic function standard curve analysis for dose response.

### Bright Field Microscopy

Colorectal cancer cells were seeded in six-well plates at a density of 4 × 10^4 ^cells per well and incubated overnight to allow the cells to adhere. The cells were treated with the listed agents and incubated for four days. The cells were washed briefly in PBS before being photographed using bright field microscopy (10× magnification). Images were taken with a Model 9.0 Monochrome-6 Camera on a computer equipped with Spot Advanced imaging software (Diagnostic Instruments Inc., Sterling Heights, MI). Three images of each treatment were taken from randomly chosen fields and a representative image was selected for display in the figure.

## Results

### New curcumin analogues are more potent than curcumin in inhibiting cell viability in human colorectal cancer cells

**Figure 1:**
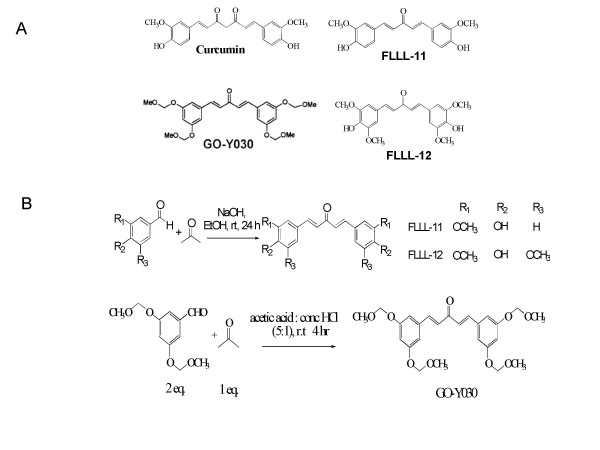
**(A) The chemical structures of curcumin, GO-Y030, FLLL-11, and FLLL-12**. (B) Synthesis of FLLL-11, FLLL-12, and GO-Y030.

**Figure 2:**
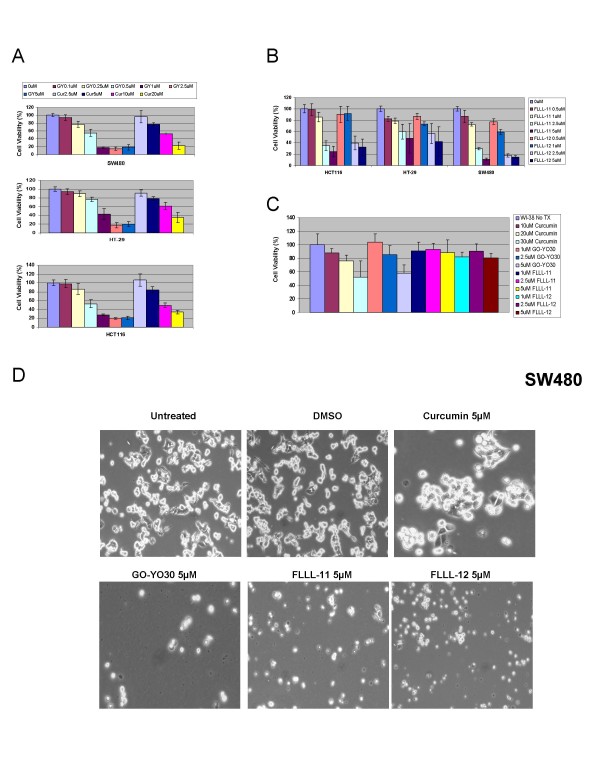
**The inhibitory effects of cell viability by (A) curcumin (Cur) and GO-Y030 (GY), (B) FLLL-11 and FLLL-12 in human colorectal cancer cell lines, HCT116, HT-29, SW480, and (C) WI-38 normal human lung fibroblasts**. Representative pictures in (D) SW480 colon cancer cells were shown.

In this study, we examined the growth suppressive activities of three structural analogues of curcumin, GO-Y030, FLLL-11, and FLLL-12 (Figures 1A and 1B), in three independent human colorectal cancer cell lines, HCT-116, SW480, and HT-29, as well as WI-38 lung fibroblasts. We first examined the inhibitory effect of cell viability by curcumin, GO-Y030, FLLL-11, and FLLL-12 in human colorectal cancer cell lines and WI-38 normal human lung fibroblasts. After 72 hours of treatment, the percent of inhibition was determined. GO-Y030, FLLL-11, and FLLL-12 all exhibited greater levels of inhibition, when compared to untreated cells in colorectal cancer cell lines than curcumin (Figures 2A and 2B). Furthermore, FLLL-11 and FLLL-12 had a low inhibitory effect on cell viability in WI-38 human lung fibroblasts (Figure 2C). Representative pictures of the inhibition of cell growth were taken for the three colorectal cancer cell lines, SW480 (Figure 2D), HCT116 and HT-29 (Data not shown). IC_50 _values were calculated for each cell line and treatment (Table 1). The results demonstrate that GO-Y030, FLLL-11, and FLLL-12 are more potent than curcumin in the growth suppression of the human colorectal cancer cell lines studied.

**Table 1:** The IC50 (μM) of curcumin and its analogues, GO-Y030, FLLL-11, and FLLL-12 in human colon cancer cells and WI-38 human lung fibroblasts

	HCT116 (IC50 μM)	HT-29 (IC50 μM)	SW480 (IC50 μM)	WI-38 (IC50 μM)
Curcumin	10.91	13.31	10.26	48.82
GO-Y030	0.61	0.96	0.51	13.78
FLLL-11	2.07	4.48	1.64	>1000.00
FLLL-12	2.49	3.35	1.17	>1000.00

*Cancer cells were treated for 72 hours and cell viability was determined by MTT assays and IC_50 _values were analyzed.

### GO-Y030, FLLL-11, and FLLL-12 are more potent than curcumin in inducing apoptosis in colorectal cancer cells

**Figure 3:**
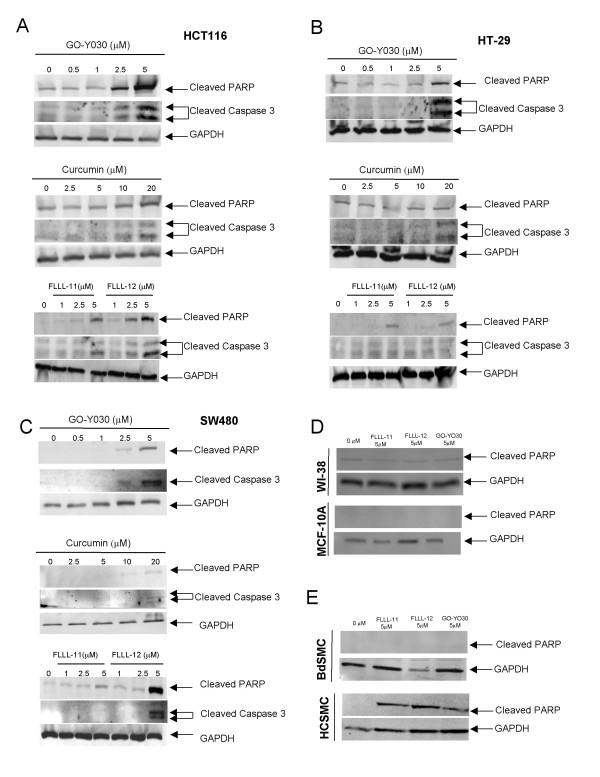
**Curcumin and its analogues, GO-Y030, FLLL-11, and FLLL-12 induced PARP and/or caspase-3 cleavages in (A) HCT116, (B) HT-29, (C) SW480 human colorectal cancer cells, (D) WI-38 and MCF-10A cells, (E) normal human and colonic bladder smooth muscle cells**. Colorectal cancer cells and normal human cells were treated for 24 hours. Western blot was used to detect the PARP cleavages using antibodies against cleaved PARP and cleaved caspase-3 (Cell Signaling Technology, Beverly, MA). The same membranes were analyzed with a 1:1000 dilution of anti-GAPDH monoclonal antibody (Chemicon International, Inc., Temecula, CA).

The induction of apoptosis was examined using cleaved PARP (Asp214) and cleaved caspase-3 (Asp175) in HCT116, SW480, and HT-29 colorectal cancer cells. GO-Y030, FLLL-11, and FLLL-12 at 2.5 and 5 μM induced cleaved PARP and cleaved caspase-3 in HCT116 colorectal cancer cells as evidenced by increased levels of cleaved PARP and cleaved caspase-3 while 10 and 20 μM of curcumin induced increased levels of cleaved PARP and caspase-3 (Figure 3A). HT-29 colorectal cancer cells seem to be the most resistance to curcumin and these three analogues, 5 μM of GO-Y030, FLLL-11, and FLLL-12 induced increased levels of cleaved PARP, whereas 10 and 20 μM of curcumin did not exhibit increased levels of cleaved PARP (Figure 3B). 5 μM of GO-Y030 and 20 μM of curcumin induced increased levels of cleaved caspase-3 while slightly increased of cleaved caspase-3 was detected in FLLL-11 and FLLL-12 treated HT-29 cells (Figure 3B). 20 μM of curcumin induced increased levels of cleaved PARP (Figure 3C). FLLL-12 at 5 and GO-Y030 at 2.5 and 5 μM induced increased levels of cleaved PARP and caspase-3 in SW480 colorectal cancer cells (Figure 3C). 20 μM of curcumin induced slightly increased levels of cleaved PARP and caspase-3 in SW480 cancer cells (Figure 3C). The results indicate that GO-Y030, FLLL-11, and FLLL-12 are more potent than curcumin in inducing PARP and caspase-3 cleavages in the three human colorectal cancer cell lines studied. Further, GO-Y030, FLLL-11 and FLLL-12 appear to induce PARP cleavages in normal human colonic smooth muscle cells. However, GO-Y030, FLLL-11, and FLLL-12 did not induce PARP cleavages in WI-38 normal human lung fibroblasts, normal human bladder smooth muscle cells, and MCF-10A non-malignant human mammary epithelial cells (Figures 3D and 3E).

## Discussion

Current anticancer therapies tend to be inadequate in terms of their therapeutic efficacy and the undesirable side effects that they produce. Bioactive compounds, known as phytochemicals, have been found to exhibit growth-suppressive activity as well as chemopreventive properties against various types of cancer [2]. Curcumin is one of the most widely characterized phytochemicals and is the active ingredient in the rhizome of the plant turmeric (*Curcuma longa*). Curcumin has both antioxidant and anti-inflammatory properties [2,3,16]. It has also been shown to protect against carcinogenesis and in the prevention of tumor formation and development in several cancer types. In a variety of animal tumor models, curcumin has been found to suppress angiogenesis and metastasis [2-6,17-21]. The growth suppressive activity and bioavailability of curcumin in humans may be effective as a preventive agent; however, it may not be sufficient as a therapeutic agent in colorectal cancer. Analogues of curcumin, which exhibit increased potency, are needed as more effective therapeutic agents for colorectal cancer treatments.

There are several curcumin analogues that have already been reported, such as dimethoxycurcumin and EF-24 [22-26]. We compared the inhibitory efficacy of new curcumin analogues, GO-Y030, FLLL-11, and FLLL-12 in colorectal cancer cell lines. We demonstrated that GO-Y030, FLLL-11, and FLLL-12 are more potent than curcumin in the inhibition of cell viability/proliferation in all three independent human colorectal carcinoma cell lines studied. GO-Y030, FLLL-11, and FLLL-12 are also more potent than curcumin in the induction of cleaved PARP, evidence of apoptosis, in the colorectal cancer cells studied. Although, cleaved PARP was also observed in normal colonic smooth muscle cells by GO-Y030, FLLL-11, and FLLL-12, GO-Y030, FLLL-11, and FLLL-12 did not induce cleaved PARP in WI-38 normal human lung fibroblasts, normal bladder smooth muscle cells, and MCF-10A immortalized, non-malignant, human mammary epithelial cells. FLLL-11 and FLLL-12 appear to be slightly less potent than GO-Y030 in the inhibition of cell viability in the colorectal cancer cell lines studied. However, FLLL-11 and FLLL-12 have less inhibitory effects than GO-Y030 in WI-38 normal human lung fibroblasts. FLLL-11 and FLLL-12 are respectively, 223 and 298 fold times more toxic in colorectal cancer cells than in WI-38 normal human lung fibroblasts compared to GO-Y030 which is only 14 fold times more toxic. Curcumin has been found to be safe in clinical trials and dose-limiting toxicity was not observed [27-30]. Similar to curcumin, FLLL-11 and FLLL-12 seem to have low toxicity in WI-38 normal human lung fibroblasts, normal bladder smooth muscle cells and non-malignant MCF-10A mammary epithelial cells except FLLL-11, FLLL-12, and GO-Y030 induce cleaved PARP in normal colonic smooth muscle cells. It will be interested to evaluate the inhibitory efficacy of FLLL-11, FLLL-12, and GO-Y030 to suppress the growth of the human colon tumor cells in mouse model *in vivo*. Further, it will also be necessary to evaluate the possible toxicity of FLLL-11, FLLL-12, and GO-Y030 comparing to curcumin in mice without tumors in vivo in particular in colon to verify the toxicity we have observed in normal colonic smooth muscle cells.

To evaluate the possible drug-likeness of these curcumin analogues, we used QikProp (Schrodinger LLC) to compute the absorption, distribution, metabolism, excretion and toxicity (ADME/Tox) properties of curcumin, FLLL-11, FLLL-12 and GO-Y030. Overall, fifty "drug-likeness" parameters have been calculated, covering molecular weight, polarity, solubility, cell permeability, blood brain barrier, HERG K^+ ^blockage, HSA binding and metabolic stability, etc. For curcumin itself, the prediction shows concern on potential HERG K^+ ^channel blockage, and more seriously a very poor cell permeability index for both Caco-2 and MDCK tests. The data seem to be consistent with the widely reported poor bioavailability of curcumin. FLLL-11, FLLL-12 and GO-Y030 all have favorable drug-likeness profiles. However, the relatively minor concerns are: 1) FLLL-11 has subpar cell permeability indexes (Caco2: 485; MDCK: 226), not as good as FLLL-12 (Caco2: 1164; MDCK: 583) and GO-Y030 (Caco2: 3073; MDCK: 1665); 2) the IC_50 _HERG K^+ ^blockage index for all three compounds are on borderline as all three compounds have QPlogHERG values around -5.5, below the -5 redline. Optimization to lessen this seems needed; 3) GO-Y030 has poor oral absorption index (index as 1 for low absorption) compared to FLLL-11 and FLLL-12 (both indexes as 3 for high oral absorption). In addition, most indicators show that GO-Y030 has the more deviations from existing drugs than FLLL-11 and FLLL-12. Overall evaluations show that FLLL-12 has the closest drug-likeness profile and has 86% similarity to propafenone and trimethobenzamide averaged over all fifty parameters. Therefore, it will be of interest to evaluate the inhibitory efficacy of these curcumin analogues in tumor model *in vivo *and *in vivo *pharmacokinetics in the future.

## Conclusion

Our results demonstrated that the curcumin analogues, GO-Y030, FLLL-11, and FLLL-12 are more potent than curcumin in the inhibition of cell proliferation and in the induction of apoptosis in three different human colorectal cancer cells. GO-Y030, FLLL-11, and FLLL-12 pass the drug-likeness test, with FLLL-12 having the highest drug-likeness. Therefore, GO-Y030, FLLL-11, and FLLL-12 have the translational potential as novel cancer therapeutic or preventive agents for human colorectal carcinoma and deserve the further evaluation.

## Abbreviations

IC_50_: Half-Maximal inhibitory concentrations; MTT: 3-(4,5-dimethylthiazol-2-yl)-2,5-diphenyltetrazolium bromide; PARP: Poly (ADP-ribose) polymerase; GAPDH: Glyceraldehyde-3-phosphate dehydrogenase; PK/PD: Pharmacokinetics/Pharmacodynamics.

## Competing interests

The authors declare that they have no competing interests.

## Authors' contributions

LC participated in experiment designs, conducted most of the experiments, contributed to the analysis and interpretation of data, and drafted the manuscript. BH and SD worked on the pictures of the treated colorectal cancer cells and contributed to the manuscript preparation. SB worked on the effects on normal human cells. CC participated in Western blots analysis. CL provided the information of drug-likeness. P-K L and J F synthesized and provided the curcumin analogues in collaboration. All authors read and approved the final manuscript.

## Pre-publication history

The pre-publication history for this paper can be accessed here:

http://www.biomedcentral.com/1471-2407/9/99/prepub
